# Peripheral Circulation and Astrocytes Contribute to the MSC-Mediated Increase in IGF-1 Levels in the Infarct Cortex in a dMCAO Rat Model

**DOI:** 10.1155/2020/8853444

**Published:** 2020-09-01

**Authors:** Xiaobo Li, Wenxiu Yu, Yunqian Guan, Haiqiang Zou, Zhaohui Liang, Min Huang, Renchao Zhao, Chunsong Zhao, Zhenhua Ren, Zhiguo Chen

**Affiliations:** ^1^Department of Neurology, Northern Jiangsu People's Hospital, Clinical Medical School of Yangzhou University, Yangzhou, China; ^2^Cell Therapy Center, Beijing Institute of Geriatrics, Xuanwu Hospital Capital Medical University, National Clinical Research Center for Geriatric Diseases, Key Laboratory of Neurodegenerative Diseases, Ministry of Education, Beijing 100053, China; ^3^Center of Neural Injury and Repair, Beijing Institute for Brain Disorders, Beijing 100069, China; ^4^Department of Neurology, The General Hospital of Guangzhou Military Command, Guangzhou, China

## Abstract

**Materials and Methods:**

Ischemic brain injury was induced by dMCAO in Sprague-Dawley rats. The transplantation group received MSC infusion 1 h after dMCAO. Expression of IGF-1 in GFAP+ astrocytes, Iba-1+ microglia/macrophages, CD3+ lymphocytes, Ly6C+ monocytes/macrophages, and neutrophil elastase (NE)+ neutrophils was examined to determine the contribution of these cells to the increase of IGF-1. ELISA was performed to examine IGF-1 levels in blood plasma at days 2, 4, and 7 after ischemia onset.

**Results:**

In total, only 5-6% of Iba-1+ microglia were colabeled with IGF-1 in the infarct cortex, corpus callosum, and striatum at day 2 post-dMCAO. MSC transplantation did not lead to a higher proportion of Iba-1+ cells that coexpressed IGF-1. In the infarct cortex, all Iba-1+/IGF-1+ double-positive cells were also positive for CD68. In the infarct, corpus callosum, and striatum, the majority (50-80%) of GFAP+ cells were colabeled with ramified IGF-1 signals. The number of GFAP+/IGF-1+ cells was further increased following MSC treatment. In the infarct cortex, approximately 15% of IGF-1+ cells were double-positive for CD3. MSC treatment reduced the number of infiltrated CD3+/IGF-1+ cells by 70%. In the infarct, few Ly6C+ monocytes/macrophages or NE+ neutrophils expressed IGF-1, and MSC treatment did not induce a higher percentage of these cells that coexpressed IGF-1. The IGF-1 level in peripheral blood plasma was significantly higher in the MSC group than in the ischemia control group.

**Conclusion:**

The MSC-mediated increase in IGF-1 levels in the infarct cortex mainly derives from two sources, astrocytes in brain and blood plasma in periphery. Manipulating the IGF-1 level in the peripheral circulation may lead to a higher level of IGF-1 in brain, which could be conducive to recovery at the early stage of dMCAO.

## 1. Introduction

Insulin-like growth factor-1 (IGF-1) is a member of the insulin gene family [1]. In addition to regulating cerebral development, neurogenesis, cognition, and memory function [2], IGF-1 is also an important player during the damage and recovery processes in ischemic stroke [3, 4].

It has been widely recognized that neuroinflammation plays a critical role in brain injuries and neurodegeneration. The role of IGF-1 in the central nervous system (CNS) is, to a large extent, due to its ability to regulate immune cells in brain, such as microglia and infiltrated macrophages.

Microglia are important players in both innate immunity and adaptive immunity. The polarization of microglia is associated with the pathogenesis of a number of inflammatory disorders, such as the acute and chronic damage after stroke. Several *in vitro* studies revealed a direct anti-inflammatory effect of IGF-1 on microglia [5, 6]. Accumulating evidence suggests that IGF-1 may also modulate microglial phenotypes; for example, an increase in IGF-1 levels promotes the switch to the M2 phenotype [7]. Macrophages can also be regulated by IGF-1. In peripheral tissues, IGF-1 impacts macrophagic functions and leads to downregulation of proinflammatory cytokines and a change in disease progression [8, 9].

Astrocytes can also produce IGF-1 and are positive for IGF-1 receptors [10, 11]. Addition of IGF-1 to the *in vitro* culture of astrocytes promotes astrocyte growth and formation of glycogen [12]. Overexpression of IGF-1 by astrocytes through an AAV-mediated delivery improves outcome in a rat stroke model [13]. Astrocyte-derived IGF-1 can also protect neurons from kainic acid- (KA-) induced excitotoxicity in an astrocyte-neuron coculture system, and the rescue effect is abrogated by adding IGF-1R inhibitor [14].

By using ELISA in a previous study, we reported an increased level of IGF-1 in the ischemic core and peri-infarct striatum in dMCAO rats at 48 h after intravenous (i.v.) infusion of rat bone marrow-derived MSCs [10]. MSC treatment leads to a higher level of IGF-1 compared to dMCAO rats without MSC infusion. By using immunostaining, we found that IGF-1 signals are mainly located in the infarct area. A minority of IGF-1 signals colocalize with NeuN+ neurons and CD68+-activated microglia in infarcts; nonetheless, quantitative analysis showed that these cells cannot account for all of the IGF-1-positive signals [15]. Other contributors in the brain and periphery (IGF-1 can cross the blood-brain barrier (BBB) [16–18]) to the increased IGF-1 signals in the brain warrant further investigation. In this study, we surveyed a wide spectrum of cell types that included Iba-1+ microglia, GFAP+ astrocytes, infiltrated immune cells such as CD3+ lymphocytes, neutrophil elastase (NE)+ neutrophils, and Ly6C+ monocytes/microphages, as well as the peripheral circulation, to determine their contribution to the increased IGF-1 level in the brain.

## 2. Materials and Methods

### 2.1. dMCAO Model

The performance of allogeneic bone marrow MSC (BMSC) culture, infusion, dMCAO model establishment, and behavioral tests have been described in our previous study [19].

In brief, primary cultures of bone marrow stromal cells were obtained from donor young adult male rats, and BMSCs were isolated as previously described [15, 19]. Animals were anesthetized with 3.5% isoflurane and then maintained with 1.0–2.0% isoflurane in N_2_O : O_2_ (2 : 1). One-hour postischemia, randomly selected rats received BMSC infusion. Approximately 1 × 10^6^ BMSCs in 1 mL of vehicle (1 mL of saline) were slowly injected over a 5 min period into each rat via the tail vein.

In total, 100 wild-type Sprague-Dawley rats were purchased from Vital River Laboratory Animal Technology (Beijing, China). All animal protocols were in accordance with the Guidelines for the Care and Use of Experimental Animals and approved by the Institutional Animal Care and Use Committee of Capital Medical University. All experimental animals were housed in a specific pathogen-free rodent barrier facility at the Xuanwu Hospital Capital Medical University, on a 12 h light : 12 h darkness cycle with food and water ad libitum.

Ten Sprague-Dawley rats were used for harvesting bone marrow MSCs. Ninety Sprague-Dawley rats were divided into three groups: “sham”, “ischemia,” and “ischemia+MSCs” with 30 rats in each group. And for each group, 3 time points—days 2, 4, and 7 postischemia—were chosen with 10 rats used for each time point.

### 2.2. IGF-1 Measurement in Blood Plasma

Blood was collected in heparinized tubes from the abdominal aorta. Subsequently, the blood samples were centrifuged at 1000×g for 10 min at room temperature, and the resulting plasma was obtained. The plasma was apportioned into 0.5 mL aliquots and stored at -80°C for further use.

IGF-1 levels were determined by ELISA using the R&D systems quantitative rat IGF-1 immunoassay kit (Minneapolis, MN, USA), which demonstrates high cross-reactivity with rat IGF-1. Plasma samples were diluted in the calibrator diluent provided in the kit at 1 : 10. The results were presented as ng/mL in undiluted plasma.

### 2.3. Brain Immunohistochemistry and Counting under Confocal Microscopy

Immunohistochemistry and cell counting were performed as previously described [15, 19, 20].

Confocal images were acquired using a Leica TCS SP5 II AOBS laser scanning confocal microscope. The section thickness was 40 μm to minimize the possibility of cell body overlap in the *z*-axis. FITC or Cy3 fluorescence was acquired with an excitation wavelength of 633 nm and detection at 648–712 nm. Cy5 was detected with an excitation wavelength of 488 nm and detection at 501–542 nm.

### 2.4. Quantification and Statistical Analysis

As described in our previous study [15, 19], the selection of sections for each rat and the demarcation of the counting area in the infarct cortex were in accordance with the work published by Gelosa et al. [21]. In brief, we outlined the counting area in the dorsal infarct cortex which was 2 mm adjacent to the boundary line between the normal and infarct areas (Supplementary Figure 1). The counting area in the striatum and corpus callosum was also demonstrated in the Supplementary Figure 1.

IGF-1-positive cells in the brain were counted in 4 coronal sections from each rat (40 *μ*m thickness, 480 *μ*m interval, located between -2.0 mm and 2.0 mm to bregma); for each slide, 2 squares of images were captured under a microscope with a view field set as 800 × 800 *μ*m (200x).

Eight fields of view were digitalized under a 20x objective, and the number of positive cells was summed for IGF-1-positive cells that coexpressed Iba-1, GFAP, CD3, Ly6C, or neutrophil elastase (NE). The proportion of all IGF-1+ cells that were double-positive for each lineage marker was calculated and averaged.

Data were presented as the mean ± standard error of the mean (SEM). The comparisons were analyzed by one-way analysis of variance (one-way ANOVA) and Bonferroni-Dunnett corrections using SPSS 19.0. The level of significance of all comparisons was set at *p* < 0.05.

## 3. Results

### 3.1. Iba-1+ Microglia/Macrophages Are Not the Main Source of IGF-1 in the Infarct Cortex

**Figure 1:**
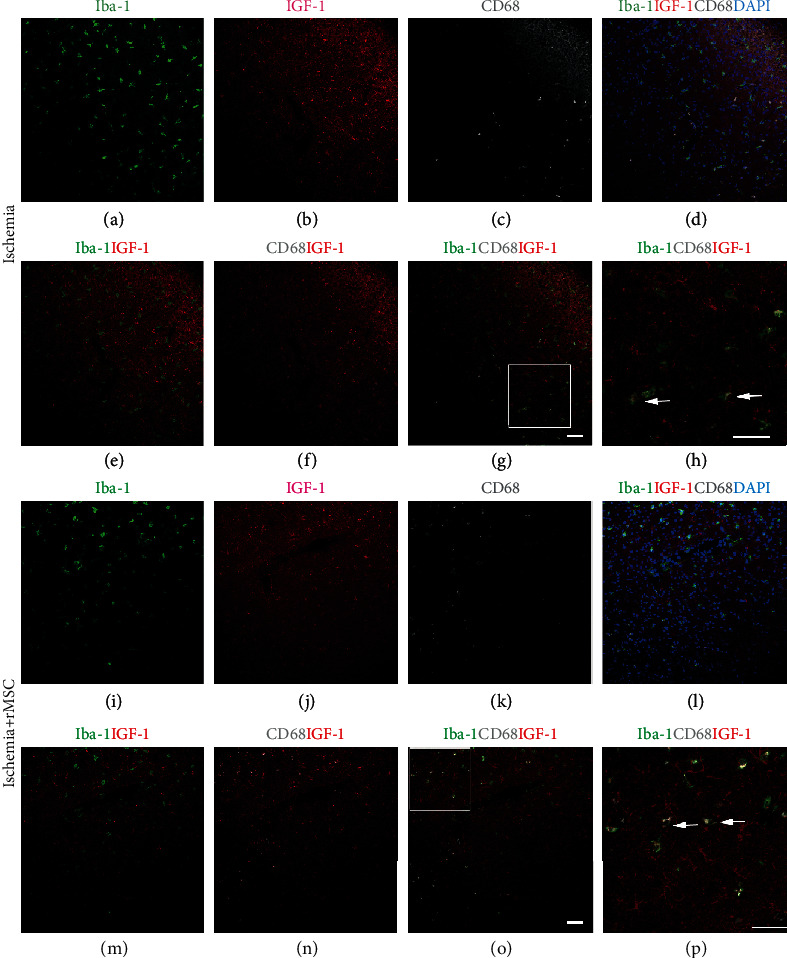
The distribution of Iba-1+, IGF-1+, and CD68+ cells in the infarct area with and without MSC infusion. (a–h) Without MSC transplantation, the distribution of IGF-1+ and Iba-1+ cells in the infarct area. (i–p) With MSC transplantation, the distribution of IGF-1+ and Iba-1+ cells in the infarct area. (a, i) Iba-1staining in green. (b, j) IGF-1 staining in red. (c, k) CD68 staining in white. (d, l) Merged image of Iba-1, IGF-1, CD68, and blue DAPI nuclear staining. (e, m) Merged image of Iba-1 and IGF-1. Very few Iba-1+/IGF-1+ cells were scattered in the infarct area. (f, n) Merged image of CD68 and IGF-1. (g, o) Merged image of Iba-1, CD68, and IGF-1. (h) (square in (g)) and (p) (square in (O)) The IGF-1/Iba-1+ cells were CD68 positive, while the CD68-/Iba-1+ cells were negative for IGF-1. Arrow: IGF-1, Iba-1, and CD68 triple-labeled cells. Scale bar, 50 *μ*m.

Microglia at an activated state vs. resting state assume different morphologies and marker expression profiles. The activated microglia stain positive for CD68 (Figure 1), and both activated and resting stage microglia are positive for Iba-1. In our previous studies, we reported that CD68+ microglia express IGF-1 in the brain of a stroke model [15, 19]. In the present study, we continued to look into a wider range of microglia population—Iba-1+ cells, with regard to IGF-1 expression.

By immunostaining, we found that IGF-1 signals were almost absent in the brain cortex in the sham group (data not shown). In the ischemia control group at day 2 after ischemia, Iba-1 and IGF-1 signals were mainly localized in the cortical infarct area, specifically the inner infarct border zone (Figures 1(a)–1(d)).

Interestingly, although the distribution patterns of Iba-1+ cells and IGF-1+ cells were similar (Figures 1(a)–1(d)), double-positive cells were only occasionally observed (Figure 1(e)).

In this study, the counts of Iba-1+/IGF-1+ cells were similar to those of CD68+/IGF-1+ cells in the infarct cortex, suggesting that IGF-1 expression in the Iba-1+ cell population might come from the CD68+/IGF-1+ double-positive cells. By triple immunostaining, we verified that the Iba-1+/IGF-1+ cells were indeed CD68+ cells (Figures 1(f)–1(h), arrow) in the infarct area of the cortex. The CD68-/Iba-1+ microglial cells were negative for IGF-1.

After MSC infusion, both IGF-1+ and Iba-1+ signals were still detected in the inner border zone of the infarct area (Figures 1(i)–1(l)). The quantities of Iba-1+/IGF+ double-positive cells, together with the percentages of Iba-1+/IGF-1+ double-positive cells among the total Iba-1+ cells or IGF-1+ cells, respectively (4.40 ± 1.49/view field, 6.93 ± 3.61%, and 8.55 ± 3.62%), were all increased compared to those of the ischemia group (2.93 ± 2.08/view field, 5.34 ± 3.93%, and 6.60 ± 4.84%).

In the infarct area, the CD68+ cells still expressed IGF-1 (Figure 1(m) and 1(n)), and the triple staining results confirmed that the Iba-1+/IGF-1+ cells were mostly CD68+ (Figures 1(o) and 1(p)). These results suggested that in the infarct area, IGF-1 expression in the Iba-1+ cell population was derived from the CD68+/IGF-1+ double-positive cells.

The quantities of Iba-1+/IGF-1+ double-positive cells in the brain infarct area, both before and after MSC infusion, were very low, indicating that Iba-1+ cells may not be the major source of IGF-1 in the ischemic cortex.

### 3.2. Iba-1+ Microglia/Macrophages Are Not the Main Source of IGF-1 in the Striatum and Corpus Callosum

In the sham group, no significant IGF-1+ signals were detected in the striatum or corpus callosum (data not shown).

**Figure 2:**
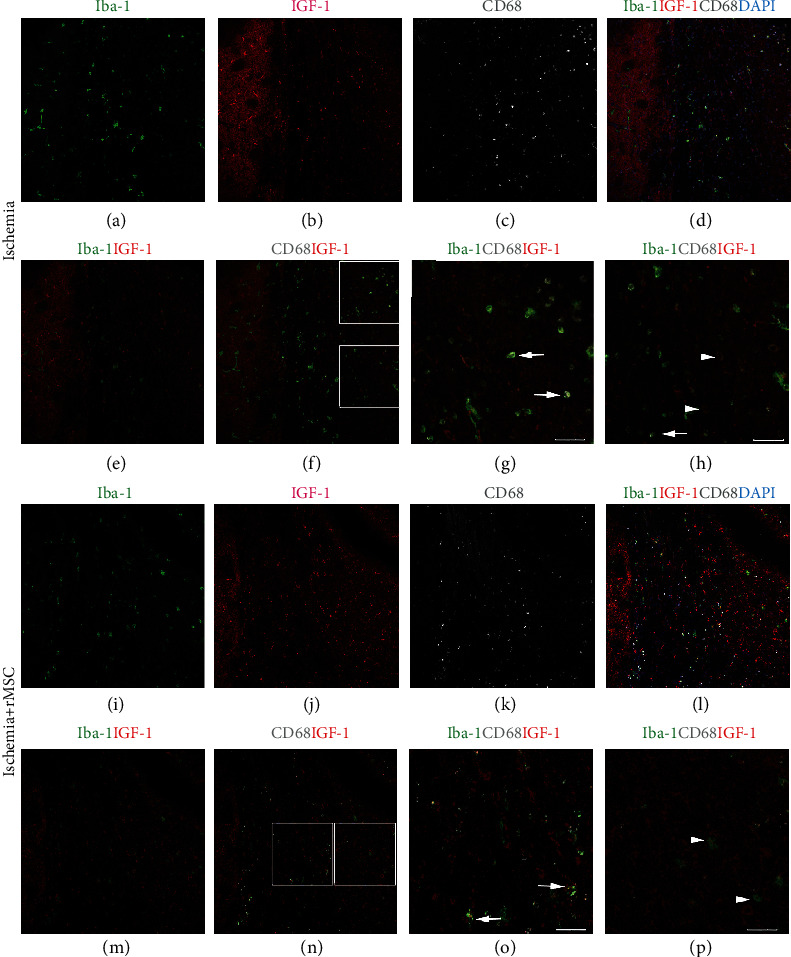
The distribution of Iba-1+, IGF-1+, and CD68+ cells in the striatum and corpus callosum with and without MSC infusion. (a–h) Without MSC transplantation, the distribution patterns of IGF-1+ and Iba-1+ cells in the corpus callosum are similar. (i–p) With MSC transplantation, the distribution of IGF-1+ and Iba-1+ cells in the corpus callosum. (a, i) Iba-1staining in green. (b, j) IGF-1 staining in red. (c, k) CD68 staining in white. (d, l) DAPI nuclear staining in blue merged with Iba-1, IGF-1, and CD68. (e, m) Merged image of Iba-1+ and IGF-1+. (f, n) Merged image of CD68, Iba-1, and IGF-1. Iba-1+/IGF-1+ double-positive cells could be found. (g, o) Merged image of Iba-1, IGF-1, and CD68. (g) (upper square in (f)) and (h) (lower square in (f)) The quantity of Iba-1+/IGF-1+ cells in the striatum and corpus callosum was not increased by MSC transplantation. The majority of Iba-1+/IGF-1+ double-positive cells were CD68+. (o) (left square in (n)) and (p) (right square in (n)) The difference in striatum vs. cortex was that IGF-1 expression was not limited to CD68+ cells. There were Iba-1+/IGF-1+ double-positive cells that were negative for CD68. Arrow: IGF-1, Iba-1, and CD68 triple-labeled cells; arrowhead: IGF-1+Iba-1+ double-positive but CD68 negative cells. Scale bar, 50 *μ*m.

Two days after ischemia onset, IGF-1+ signals were found scattered in the striatum and corpus callosum (Figures 2(a)–2(d)). Although the distribution patterns of Iba-1+ cells and IGF-1+ cells were similar (Figures 2(a)–2(d) and Figures 2(e)–2(h)), the quantity of Iba-1+/IGF-1+ double-positive cells remained very low (4.53 ± 2.03/view field) (Figures 2(g)–2(h)). The percentages of Iba-1+/IGF-1+ cells among the total Iba-1+ cells or IGF-1+ cells were 7.83 ± 3.50% and 9.22 ± 4.62%, respectively.

CD68+ cells represent an activated subpopulation of microglia and were detected to be positive for IGF-1 (Figures 2(e) and 2(f)). The expression patterns of IGF-1 in microglia differed in striatum and corpus callosum vs. in infarct cortex; in striatum and corpus callosum, Iba-1+/IGF-1+ double-positive cells were not limited to CD68+ cell population, and 30-40% of the Iba-1+/IGF-1+ double-positive cells stained negative for CD68 (Figures 2(g) and 2(h)).

In the striatum and corpus callosum, neither the distribution patterns of IGF-1+ and Iba-1+ cells (Figures 2(i)–2(l)) nor the quantity of double-labeled cells (5.40 ± 1.96/view field) was significantly changed by MSC treatment (Figures 2(m) and 2(n)). The double-positive cells were highlighted in a magnified view (Figures 2(o) and 2(p)). In addition, MSC infusion did not change the percentages of Iba-1+/IGF-1+ double-positive cells among the total Iba-1+ (9.92 ± 5.21%) or IGF-1+ cells (11.39 ± 4.59%).

Taken together, Iba-1+/IGF-1+ cells constituted a small part of the IGF-1+ cells in the infarct area, striatum, and corpus callosum, suggesting that the Iba-1+ cell population is not the main source of IGF-1.

### 3.3. GFAP+ Astrocytes Are the Main Cell Source of IGF-1 in Infarct Area

Previously, we found that in the ischemia brain cortex of dMCAO model, IGF-1 was partially expressed by NeuN+ neurons and CD68+ microglia/macrophages. On top of that, a significant number of other IGF-1-expressing cells existed, which included cells with a ramified morphology, reminiscent of GFAP+ astrocytes.

Due to the similarity in the morphology and distribution pattern of IGF-1+ signals and GFAP+ astrocytes in the ischemic hemisphere, double fluorescent immunostaining was employed to examine the spatial relationship of these two markers.

**Figure 3:**
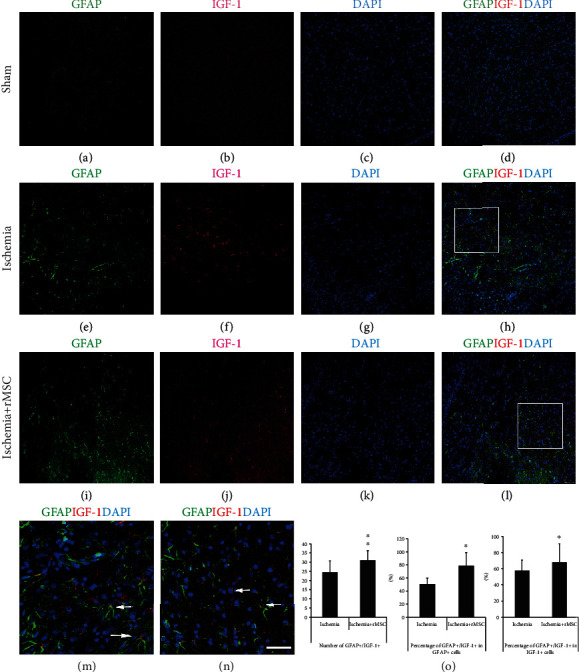
MSC treatment reduces the quantity of GFAP+ cells but increases the number of IGF-1+/GFAP+ cells in the infarct. (a–d) In the sham group, a few GFAP+ cells could be found in the brain cortex. (e–h) In the infarct area of the ischemia control group, the number of GFAP+ and IGF-1+ cells both increased. (i–l) In the infarct area, MSC treatment decreased GFAP+ cell quantity but increased the number of cells double positive for GFAP and IGF-1. (a, e, and i) GFAP staining. (b, f, and j) IGF-1. (c, g, and k) DAPI nuclear staining. (d, h, and l) Double-stained GFAP+/IGF-1+ cells. (m, n) (squares in (h, l) The amplified view of GFAP+/IGF-1+ cells. (o) After MSC transplantation, the quantity of GFAP+/IGF-1+ double-positive cells and the percentages among all IGF-1+ or GFAP+ cells increased. Scale bar, 50 *μ*m.

As shown in Figure 3, an increased level of astrogliosis was observed 48 h following dMCAO (Figures 3(e)–3(h)) compared to sham controls (Figures 3(a)–3(d)). Following MSC treatment, the number of GFAP+ astrocytes (42.3 ± 5.2/view field) was slightly but significantly reduced compared to that in the ischemia control group (48.43 ± 9.67/view field). However, the quantity of GFAP+/IGF-1+ double-positive cells in the infarct area increased following infusion of MSCs (Figures 3(i)–3(l)) from 24.47 ± 6.17/view field in the ischemia vehicle group to 31.03 ± 5.22/view field (*p* < 0.01) in the MSC group. The double-positive cells were highlighted in a magnified view (Figures 3(m) and 3(n)). As a net result, the percentage of GFAP+/IGF-1+ double-positive cells among the GFAP+ astrocytes was significantly increased from 50.79 ± 8.97% in the ischemia control group to 78.70 ± 20.11% in the MSC group (Figure 3(o)). This suggested that MSC treatment had induced more astrocytes to express IGF-1.

The above data suggested that GFAP+ astrocytes present in the infarct area of dMCAO rats were the main source of IGF-1, since 57.64 ± 12.93% of IGF-1+ cells in the ischemia group and 68.44 ± 23.42% in the MSC infusion group were GFAP+ astrocytes (Figure 3(o)).

### 3.4. GFAP+ Astrocytes Are the Main Source of IGF-1 in the Striatum and Corpus Callosum

**Figure 4:**
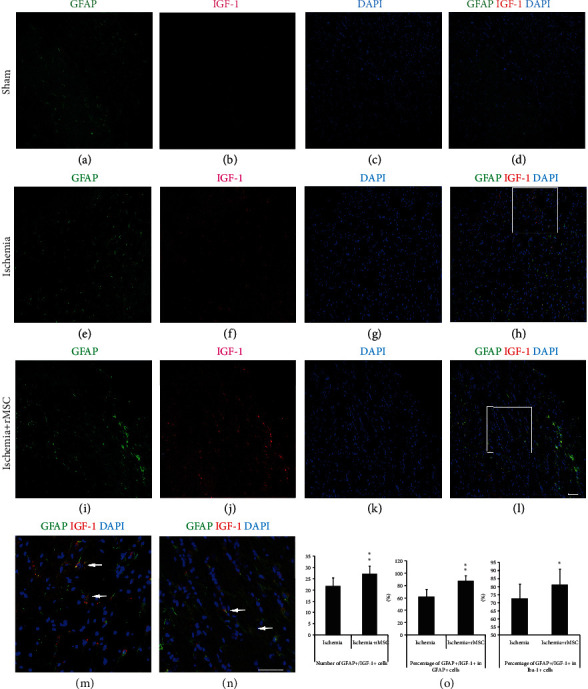
MSC treatment decreases the quantity of GFAP+ cells but increases GFAP+/IGF-1+ cells in the ipsilateral striatum and corpus callosum. (a–d) In the striatum and corpus callosum of the sham group, IGF-1 signals were rarely found. (e–h) In the striatum and corpus callosum of the ischemia control group, both IGF-1 and GFAP signals were increased. (i–l) After MSC transplantation, more GFAP+/IGF-1+ cells were observed in the striatum and corpus callosum. (a, e, and i) GFAP staining. (b, f, and j) IGF-1. (c, g, and k) DAPI nuclear staining. (d, h, and l) Double-stained GFAP+/IGF-1+ cells. (m, n) (squares in (h, l)) The amplified view of GFAP+/IGF-1+ cells. (o) The histogram of GFAP+/IGF-1+ cell quantity and percentages in the ischemia group and MSC transplantation group (^∗^*p* < 0.05, ^∗∗^*p* < 0.01, compared with the ischemia vehicle group). Arrow: double-labeled cells. Scale bar, 50 *μ*m.

In addition to the infarct cortex, numerous GFAP+ astrocytes and a few IGF-1+ cells were detected in the striatum and corpus callosum of sham group (Figures 4(a)–4(d)).

Compared to the ischemia control group (Figures 4(e)–4(h)), MSC infusion induced a slightly but significantly reduced number of GFAP+ astrocytes (from 36.4 ± 9.72/view field to 31.4 ± 4.54/view field) (Figures 4(i)–4(l)), but an increased number of GFAP+/IGF-1+ double-positive cells (from 21.7 ± 3.78/view field to 27.27 ± 3.43/view field) (Figures 4(i)–4(l), and 4(o)). The double-positive cells were highlighted in a magnified view (Figures 4(m) and 4(n)). The proportion of astrocytes that coexpressed IGF-1 was enhanced from 62.20 ± 12.12% to 87.45 ± 8.64%.

GFAP+ astrocytes present in the striatum and corpus callosum might be the main sources of IGF-1 expression in that area, in that GFAP+/IGF-1+ double-positive cells constituted up to 72.66 ± 8.98% of IGF-1+ cells in the ischemia group, which was significantly increased to 81.22 ± 9.70% in the MSC group (Figure 4(o)).

### 3.5. CD3+/IGF-1+ Cells Increase Significantly after Ischemia and Are Decreased with MSC Treatment

**Figure 5:**
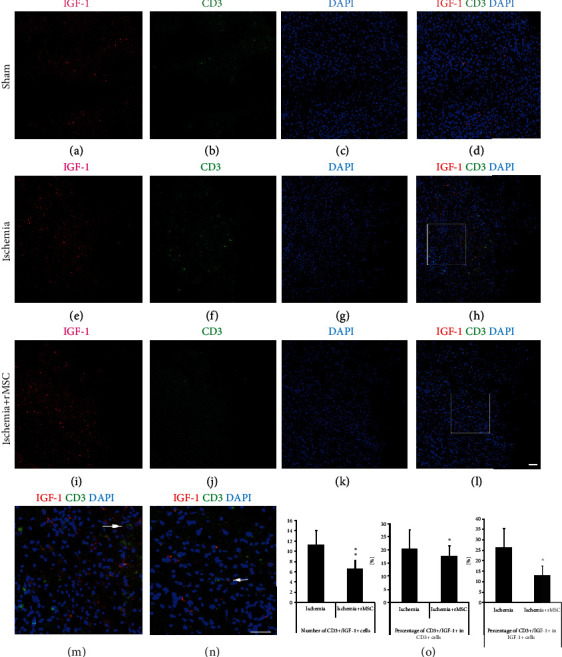
CD3+ cells that express IGF-1 in the infarct area are reduced by MSC treatment. (a–d) In the sham group, CD3+ lymphocytes were barely observed in the brain. (e–h) CD3+ and CD3+/IGF-1+ cells increased significantly in the infarct area of the ischemia control group. (i–l) The number of CD3+ and CD3+/IGF-1+ cells in the infarct area was reduced dramatically by MSC transplantation. (a, e, and i) CD3 staining. (b, f, and j) IGF-1. (c, g, and k) DAPI nuclear staining. (d, h, and l) Double-stained CD3+/IGF-1+ cells. (m, n) (insets in (h, l)) The amplified view of CD3+/IGF-1+ cells in the ischemia and MSC transplantation groups. (o) The histogram of CD3+/IGF-1+ cell quantity and percentages in the ischemia group and MSC transplantation group (^∗^*p* < 0.05, ^∗∗^*p* < 0.01, compared with the ischemia vehicle group). Arrow: double-labeled cells. Scale bar, 50 *μ*m.

In the brain cortex of the sham group, few CD3+ lymphocytes were detected (Figures 5(a)–5(d)). In the ischemia control group, a markedly larger number of CD3+ cells were observed in the infarct area (Figures 5(e)–5(h)). Double staining revealed that CD3+/IGF-1+ cells were mainly distributed in the inner border zone of the infarct area (Figures 5(e)–5(h)).

MSC treatment reduced the quantity of CD3+/IGF-1+ cells that had infiltrated into the brain from 11.20 ± 2.95 per view field in the ischemia group to 6.6 ± 1.28 in the MSC group (Figures 5(i)–5(l)). The double-positive cells were highlighted in a magnified view (Figures 5(m) and 5(n)). MSC treatment also reduced the proportion of CD3+ cells that coexpressed IGF-1 from 35.62 ± 14.46% before MSC treatment to 27.54 ± 7.86% after treatment (Figures 5(i)–5(l) and 5(o)).

In the ischemia group, the percentage of CD3+/IGF-1+ cells among IGF-1+ cells was 26.46 ± 8.91%, which was dramatically reduced by MSC infusion to 13.14 ± 4.38% (Figures 5(i)–5(l) and 5(o)).

### 3.6. Limited Contribution from Ly6C+ Monocytes/Macrophages to IGF-1 Expression

In our previous study, we reported that both Ly6C+ infiltrated monocytes/macrophages and brain-derived neurotrophic factor (BDNF) immunostaining are mainly located in the infarct boundary zone. The Ly6C+ cells are the main source of BDNF [19]. In the present study, we continued to investigate the contribution of infiltrated Ly6C+ cells to IGF-1 expression.

**Figure 6:**
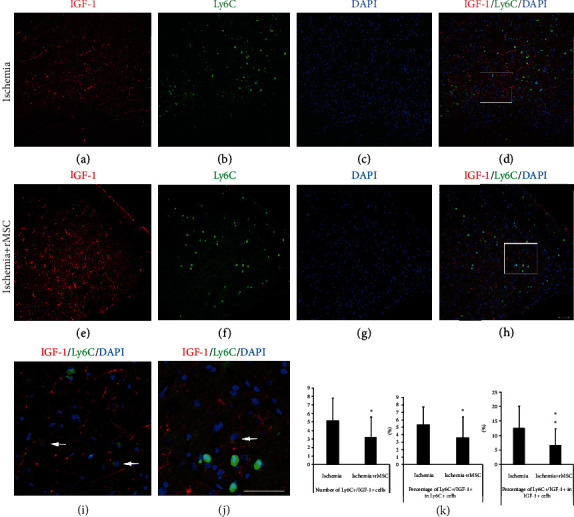
MSC treatment reduces the number of infiltrated Ly6C+ cells that express IGF-1. (a–d) In the ischemia control group, Ly6C+ and Ly6C+/IGF-1+ cells were mainly located in the infarct areas. The quantity of Ly6C+ cells that expressed IGF-1 in the ischemia core cortex of the control brain was very small. (e–h) After MSC infusion, the distribution of Ly6C+ and IGF-1+ cells was still restricted in the infarct area of the ipsilateral cortex. MSC infusion reduced the quantity of Ly6C+ cells infiltrated into the brain and decreased the percentage of Ly6C+ cells that coexpressed IGF-1. (a, e) IGF-1 staining. (b, f) Ly6C staining. (c, g) DAPI nuclear staining. (d, h) Double-stained Ly6C+/IGF-1+ cells. (i) (inset in (d)) In the inner infarct boundary zone of the ischemia group, the amplified view of Ly6C+/IGF-1+ cells. (j) (inset in (h)) In the MSC transplantation group, the amplified view of Ly6C+/IGF-1+ cells. (k) The histogram of Ly6C+/IGF-1+ cell quantity and percentages in the ischemia group and MSC transplantation group (^∗^*p* < 0.05, ^∗∗^*p* < 0.01, compared with the ischemia vehicle group). Arrow: Ly6C+/IGF-1+ double-labeled cells. Scale bar, 50 *μ*m.

At day 2 after dMCAO, Ly6C+ infiltrated monocytes/macrophages were found mainly in the inner border zone of the infarct. Ly6C+ signals were scattered around but rarely colocalized within the cytoplasm of IGF-1-expressing cells (Figures 6(a)–6(d)). In the inner infarct border zone, there were 5.20 ± 2.63 Ly6C+/IGF-1+ cells per view field; only 5.38 ± 2.34% of Ly6C+ cells coexpressed IGF-1, and 12.67 ± 7.72% of IGF-1+ cells coexpressed Ly6C+ (Figures 6(a)–6(d) and 6(k)).

MSC treatment not only reduced the number of Ly6C+/IGF-1+ cells to 3.20 ± 2.43/view field but also decreased the percentage of Ly6C+/IGF-1+ cells among the Ly6C+ population to 3.64 ± 2.78% (Figures 6(e)–6(h)). The double-positive cells were highlighted in a magnified view (Figures 6(i) and 6(j)). The percentage of Ly6C+/IGF-1+ cells among the IGF-1+ cells decreased accordingly to 6.71 ± 5.74% (Figures 6(e)–6(h) and 6(k)).

Taken together, these results indicated that only a small portion of Ly6C+ monocytes/macrophages were capable of expressing IGF-1, and Ly6C+ cells in this experimental setting were probably not the main cellular source of IGF-1 expression.

### 3.7. NE+ Neutrophils Do Not Express IGF-1

**Figure 7:**
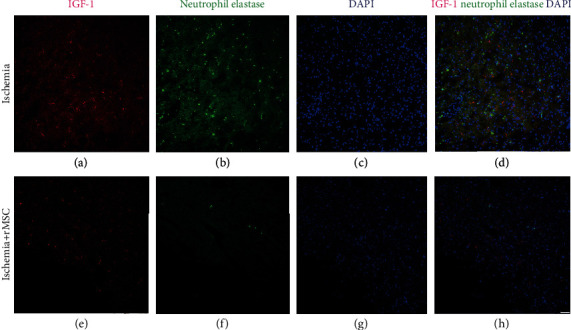
NE+ neutrophils in the ischemia core cortex are not the main source of IGF-1. (a–d) In the infarct boundary zone of the ischemia group, immunohistological examination showed strong expression of neutrophil elastase (NE), a marker for neutrophils, mainly located inside the entire cerebral ischemia core cortex but not in the striatum or corpus callosum. No double staining of NE+/IGF-1 cells were found. (e–h) NE+ cell number was reduced in the infarct area 2 days after MSC transplantation. No double-stained NE+/IGF-1+ cells were observed in the transplantation group. (a, e) IGF-1 staining. (b, f) Neutrophil elastase staining. (c, g) DAPI nuclear staining. (d, h) Merged images of neutrophil elastase, IGF-1, and DAPI. Scale bar, 50 *μ*m.

Few NE+ neutrophils were detected in the brains of the sham group (data not shown). In the dMCAO model, NE+ neutrophils existed in both the ischemia control group (Figures 7(a)–7(d)) and the MSC treatment group (Figures 7(e)–7(h)). However, no NE+ neutrophils coexpressing IGF-1 were observed, suggesting that NE+ neutrophils were not the source of IGF-1 in this experimental setting.

### 3.8. The Plasma Level of IGF-1 Is Increased following MSC Treatment

**Figure 8:**
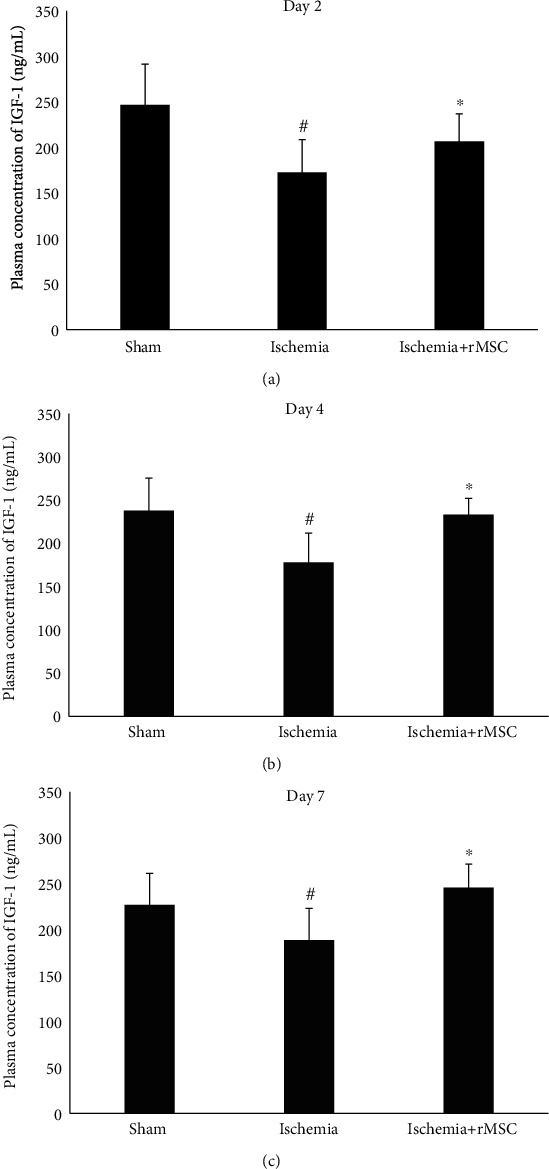
IGF-1 plasma levels at days 2, 4, and 7 after dMCAO and MSC infusion. (a–c) Two, 4, and 7 days after MSC infusion.

The concentration of IGF-1 in the blood plasma (172.7 ± 35.9 ng/mL) of ischemia group was lower than that of sham group (246.9 ± 44.5 ng/mL). Forty-eight hours following MSC treatment in dMCAO rats, the IGF-1 level in blood plasma was significantly augmented to 206.5 ± 30.3 ng/mL (Figure 8(a)).

Similarly, the concentrations of IGF-1 in the ischemia group were 178.2 ± 33.5 ng/mL and 188.7 ± 34.5 ng/mL at days 4 and 7, respectively, which were significantly increased following MSC treatment to 233.1 ± 18.9 ng/mL (*p* < 0.05) and 245.8 ± 25.7 ng/mL, respectively (*p* < 0.05) (Figures 8(b) and 8(c)).

## 4. Discussion

IGF-1 has been implicated as a potential neuroprotective agent in hypoxia-ischemia-induced damage [20, 22]. Previously, by using ELISA on nonperfused brain tissues, we reported that IGF-1 concentrations increase in ischemia core and striatum in dMCAO model at 48 h after MSC infusion; yet levels of other neurotrophic factors, such as glial cell line-derived neurotrophic factor (GDNF) and vascular endothelial grown factor (VEGF) are not changed compared to those of the ischemia vehicle group at 2, 4, and 7 days after ischemia onset and MSC infusion [15].

In the previous study, we reported that IGF-1 signals are mainly (∽60%) located in the cortex infarct area. A smaller proportion (∽40%) of IGF-1 signals are detected in the striatum and corpus callosum. In the infarct area, IGF-1 signals are colocalized with NeuN and CD68, the markers of neurons and activated microglia/macrophages, respectively; but all of these cells together only contribute to a small part of the entire IGF-1+ signals [15]. These results strongly suggest that there are other cell types as the source(s) of IGF-1 in the ischemia ipsilateral hemisphere. In the present study, we examined additional possible sources in the brain and periphery and found that astrocytes and peripheral circulation may be the main contributors to the increased level of IGF-1.

Due to the autofluorescence problem caused by blood cells, brain section staining is usually performed on transcardially perfused animals. IGF-1 is a factor that can readily cross the BBB. The consequence of perfusion is that IGF-1 diffused in the CSF and extracellular matrix would have been washed away, and only intracellular IGF-1 could be detected by staining on brain sections. This caveat may lead to an underrepresentation of IGF-1+ signals on perfused brain sections.

Upon brain injury, astrocytes are activated, which leads to phagocytosis, antigen processing and presentation, and the production of both neurotrophic and cytotoxic factors. Astrocytes are also a heterogeneous population, and reactions of astrocytes may depend on the nature of the activating stimulus and the microenvironment.

During ischemic injury, astrocytes were activated and upregulated expression of IGF-1 (Figures 3 and 4). MSC treatment induced astrocytes to further enhance IGF-1 expression without increasing the number of activated astrocytes (Figures 3 and 4). The molecular cues that promoted astrocytic expression of IGF-1 remained to be addressed.

Astrocytes are considered to play dual functions in neuroinflammation. On the one hand, astrocytes can produce neurotrophic factors including nerve growth factor (NGF), BDNF, and IGF-1 [23]. On the other hand, activated astrocytes may release cytotoxic factors, such as reactive oxygen and nitrogen species (superoxide anion, nitric oxide) and toxic amino acids [24]. It was proposed by some researchers that astrocytes could be categorized into polarized A1 and alternatively A2 status [25]. Yet how MSC treatment may impact this A1 vs. A2 balance remains unknown and warrants further investigation.

In the present study, a majority (50-80%) of GFAP+ astrocytes were IGF-1+. Upregulation of IGF-1 in the brain may further facilitate astrocytes to generate IGF-1. IGF-1 gene therapy can reduce the reactivity of astrocytes in response to proinflammatory stimuli *in vitro* [26] and exert neuroprotective and neuroreparative actions in experimental animal models of hypoxia and stroke [27–29]. Our results suggest that IGF-1 may mediate a positive feedback loop in the regulation of astrocytes. Further studies are needed to investigate the detailed mechanisms underlying the interaction between astrocytes and IGF-1 in dMCAO models.

IGF-1 is able to cross the BBB; therefore, it is important to examine the peripheral levels of IGF-1 as well. The primary site of IGF-1 production in peripheral organs is the liver, but IGF-1 is not required for postnatal body growth in mice [30]. Blood-derived IGF-1 was increased by MSC treatment at days 2, 4, and day 7 in this study. The results suggest that MSC treatment-mediated changes of IGF-1 levels in blood may be an important mechanism leading to an increased level of IGF-1 in the brain. In some studies where MSC was infused through an intravenous route, no MSCs are observed in brain but the therapeutic and neuroprotective effects remain obvious, implying an indirect function through peripheral mediators [31].

It is unclear how MSC infusion induces upregulation of IGF-1 in the periphery and whether this increase of IGF-1 results from a direct effect of MSCs on hepatocytes. If manipulation of circulating IGF-1 levels at the acute phase of dMCAO can affect disease outcome, future therapeutic strategies may be contemplated in this direction for treatment of stroke.

## Data Availability

The data used in the manuscript supporting the conclusions of the study can be accessed by email request.

## Abbreviations

BBB: Blood-brain barrier
BDNF: Brain-derived neurotrophic factor
dMCAO: Distal middle cerebral artery occlusion
FGF2: Fibroblast growth factor-2
GDNF: Glial cell line-derived neurotrophic factor
IGF-1: Insulin-like growth factor-1
KA: Kainic acid
NE: Neutrophil elastase
NGF: Nerve growth factor
VEGF: Vascular endothelial grown factor.

## Supplementary Materials

Supplementary Materials Supplementary Figure 1: schematic demonstration of the counting areas. The cerebral infarct area from a rat in the “ischemia control” group was indicated by staining with triphenyltetrazolium chloride (TTC). Rectangles indicate the areas where cell counting was performed.
