# Supplementary material for: Peripheral Circulation and Astrocytes Contribute to the MSC-Mediated Increase in IGF-1 Levels in the Infarct Cortex in a dMCAO Rat Model
Source: Stem Cells Int. 2020 Sep 1;2020:8853444. doi: 10.1155/2020/8853444 (PMC7481998; doi:10.1155/2020/8853444)
Supplement: Supplementary Materials — Supplementary Figure 1: schematic demonstration of the counting areas. The cerebral infarct area from a rat in the “ischemia control” group was indicated by staining with triphenyltetrazolium chloride (TTC). Rectangles indicate the areas where cell counting was performed. [file 8853444.f1.docx]

**Supplementary Materials**


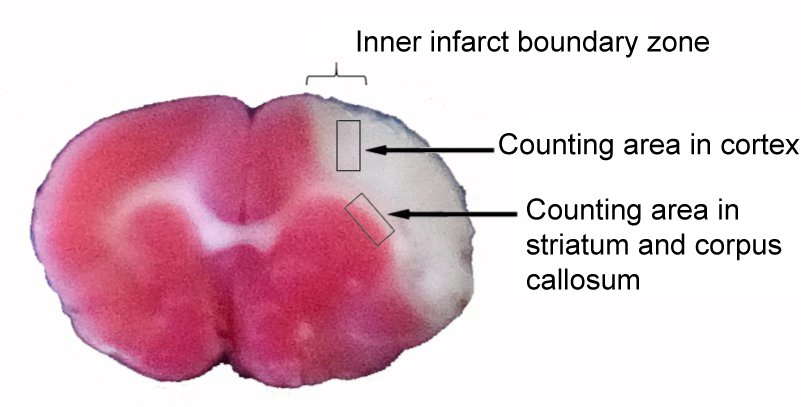


**Supplementary Figure 1. Schematic demonstration of the counting areas.**

The cerebral infarct area from a rat in the “ischemia control” group, was indicated by staining with triphenyltetrazolium chloride (TTC). Rectangles indicate the areas where cell counting was performed. Scale bar, 50 μm.
